# The worldview of *Akkermansia muciniphila*, a bibliometric analysis

**DOI:** 10.3389/fmicb.2025.1500893

**Published:** 2025-03-04

**Authors:** Zhao Zhang, Jingyu Wang, Shaoqing Dang, Xingzi Liu, Yuemiao Zhang, Hong Zhang

**Affiliations:** ^1^Renal Division, Peking University First Hospital, Beijing, China; ^2^Institute of Nephrology, Peking University, Beijing, China; ^3^Key Laboratory of Renal Disease, Ministry of Health of China, Beijing, China; ^4^Key Laboratory of Chronic Kidney Disease Prevention and Treatment (Peking University), Ministry of Education, Beijing, China; ^5^Research Units of Diagnosis and Treatment of Immune-mediated Kidney Diseases, Chinese Academy of Medical Sciences, Beijing, China

**Keywords:** *Akkermansia muciniphila*, probiotics, bibliometric analysis, Innography, research progress, patent transformation, therapeutic potential

## Abstract

*Akkermansia muciniphila* (*A. muciniphila*), a critical bacterium within the gut microbiota, plays a key role in human health and immunomodulation. Since its identification in 2004, *A. muciniphila* has emerged as a significant agent in treating metabolic diseases, gastroenterological diseases, and tumor immunotherapy. Its rapid ascent in scientific translation underscores its importance in gut microbiome research. However, there has been a lack of visualization and analysis of the rapidly occurring commercialization in this field, which has critically hindered insights into the current knowledge structure and understanding of the cutting-edge of the discipline. This study employs the Web of Science Core Collection (WOSCC) and Innography platforms to provide the first comprehensive analysis of *A. muciniphila*’s academic progresses and commercialization over the past two decades, highlighting its growing prominence in global health research. Our analysis delineates that, following the academic trajectory, the evolution of *A. muciniphila* patents from foundational research through to application development and maturity, with particular emphasis on its expansive potential in emerging fields, including gastroenterological disorders, non-alcoholic fatty liver disease, cancer immunotherapy, stress management, and neurodegenerative disease treatment. Concluding, *A. muciniphila* presents as a next-generation probiotic with vast implications for human health. Our findings provide essential insights for future research and product development, contributing to the advancement of this burgeoning field.

## 1 Introduction

There is no dispute about the fact that the interaction between the gut microbiota and human health status and immune regulation has become increasingly clear, and its depth is beyond imagination. In-depth exploration in this field continues to drive groundbreaking original research, allowing us to gain a full understanding of the gut microbiota and retrieve information about them in multiple databases. In addition, projects led and initiated by some forward-thinking research teams, such as the US Human Microbiome Project and the European Human Gut Microbiome Project, are continuously deepening our understanding of gut microbiota (Lupp et al., 2012). The achievements of these projects not only expand our knowledge of gut microbiota, but also open a new scientific perspective: that the human body and microorganisms do not exist in isolation, but are a symbiotic life community. These findings not only challenge our traditional understanding of life, but also pave the way for future research, leading the scientific community to explore the symbiotic relationship between the human body and microorganisms more deeply.

In this vast research landscape, the connection between *A. muciniphila* and the macroscopic disease health system of all humanity is undoubtedly the most dazzling one in recent years. This bacterium, which was first identified in 2004 (Derrien et al., 2004), has already reached a research height that most of its counterparts have struggled to reach in the past 20 years. Research has increasingly highlighted its potential as a beneficial gut commensal out of its symbiotic ability to degrade mucus and the capacity to improve intestinal barrier function, which is fundamental to many autoimmune diseases and chronic conditions (Derrien et al., 2004; Cani et al., 2022; Cani and de Vos, 2017). Numerous studies have shown a negative association between fecal levels of *A. muciniphila* and diseases such as inflammatory bowel diseases (IBD) (Pittayanon et al., 2020; Png et al., 2010; Rajilić-Stojanović et al., 2013; Zhang et al., 2020), obesity (Dao et al., 2016; Everard et al., 2013; Plovier et al., 2017), type 2 diabetes (Fassatoui et al., 2019), cardiometabolic disorders (Han et al., 2022; Segers and de Vos, 2023), asthma (Demirci et al., 2019), and type 1 diabetes (Fassatoui et al., 2019; Dehghanbanadaki et al., 2020; Hänninen et al., 2018). Specifically, *A. muciniphila* exerts its beneficial effects through the production of extracellular and secreted proteins, metabolites, and cell envelope components, which have been studied for their structure, signaling capacity, and effects in preclinical models. What is even more remarkable is that *A. muciniphila* has quickly integrated into the wave of scientific translation. Indications suggest that the rapidly emerging research in the academic community is defining *A. muciniphila* as the next generation probiotic at a rapid pace. However, *A. muciniphila* only accounts for 1–4% of the total gut microbiota (Geerlings et al., 2021; Karcher et al., 2021). This clever juxtaposition highlights the need for human exploration and domestication of *A. muciniphila*. Although this juxtaposition is largely an unintended outcome of the accumulation of massive research, at its essence, it is because of the guiding nature of *A. muciniphila* as a research focus. Despite significant progress, numerous research gaps persist in our understanding of *A. muciniphila*. The intricate mechanisms responsible for its health benefits continue to be unraveled. Further investigation is essential to comprehend the factors that influence its abundance and functional diversity among different individuals. While several promising applications have explored the use of *A. muciniphila* in treating diseases, additional research is imperative to effectively translate this potential into therapeutic interventions.

Bibliometrics is a discipline that was established independently in 1969, based on the measurement of network information (Mou, 2019). It can quantitatively measure the knowledge structure and citation landscape of a particular research field, providing a reliable and credible method for analyzing research patterns and assessing research progress. To date, there has been a lack of bibliometric analysis on *A. muciniphila*. More importantly, no studies have yet visualized and analyzed the rapidly occurring commercialization in this field. These limitations have hindered insights into the knowledge structure of this field from both academic performance and commercialization perspectives. In this study, we conducted bibliometric analysis and visualization of relevant academic publications and patent documents using two platforms, WOSCC and Innography. This comprehensive review of the 20 years of academic progress and commercial landscape in this field aims to effectively address the limitations and provide important references for more comprehensive research in both basic science and clinical applications.

## 2 Methods

### 2.1 Data sources

To gather the necessary data for our study, we utilized two main sources: WOSCC database for publication sources and the Innography database for patent sources.

We opted for the WOSCC database as our publication source, a decision that has been extensively explained in our previous research (Wang et al., 2023, 2024a,b,c; Pan et al., 2024; Sun et al., 2024; Chen et al., 2025). In essence, we chose this database due to its comprehensiveness, inclusion of citation reports, availability of downloadable datasets containing reference information, and its adherence to Bradford’s Law and Garfield’s Law. These factors collectively ensure the credibility of our study by guaranteeing accuracy and capturing core publications comprehensively.

For patent sources, we opted for the Innography database. This internationally recognized high-end patent search and analysis platform offers access to over 100 million patent data, more than 9 million non-patent scientific and technical documents, and information on over 7 million trademarks. With its extensive coverage, the Innograph database stands as one of the most comprehensive patent databases available. Moreover, Innography offers a patent evaluation system that aids in analyzing patent competitiveness, transformation, citations, hot trends, and other valuable insights.

### 2.2 Search strategy and keyword cleansing

**Figure 1:**
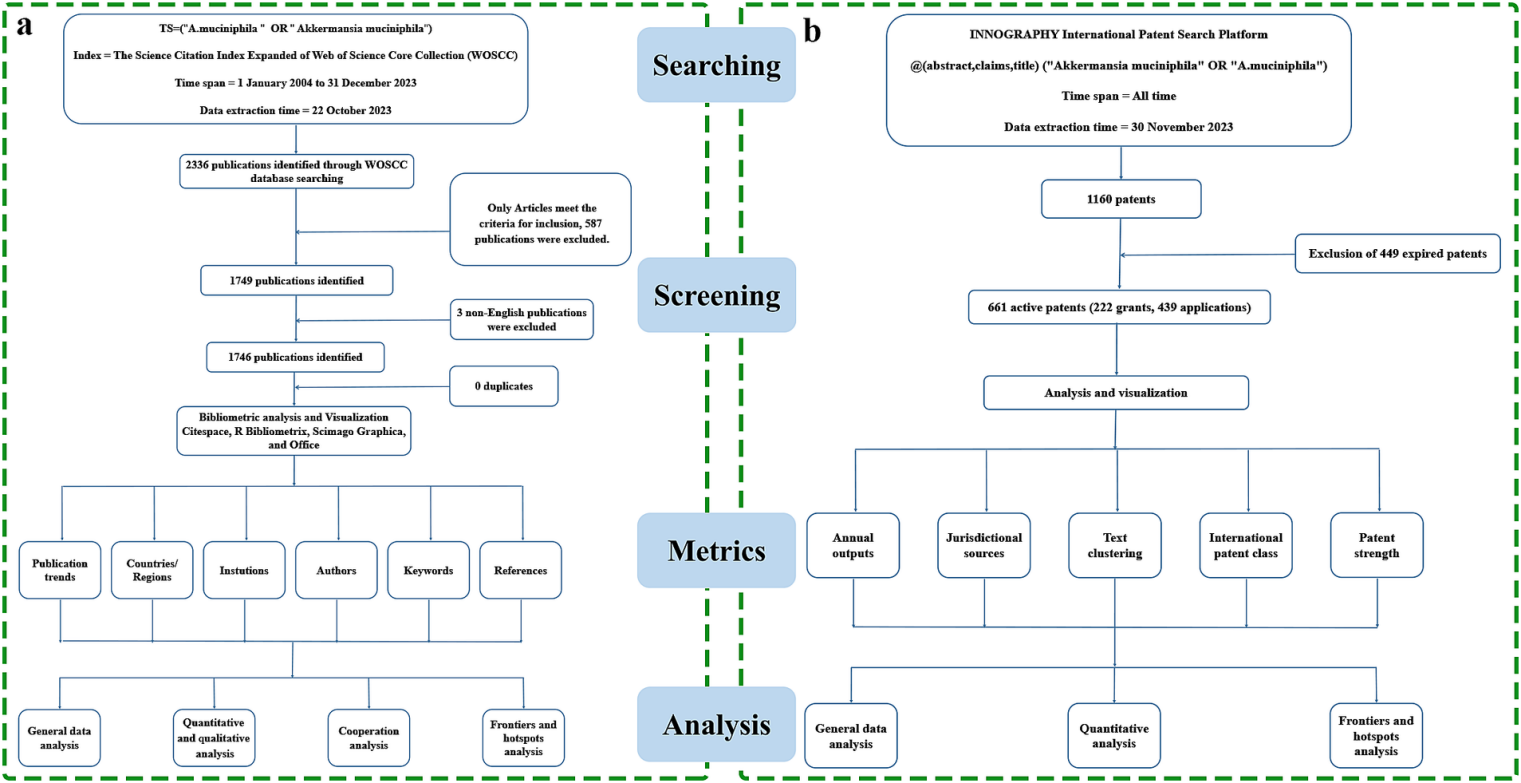
**(A)** Publications screening flowchart. **(B)** Patents screening flowchart.

The terms “*Akkermansia muciniphila*” and “*A. muciniphila*” encompass our research topic comprehensively (Dehghanbanadaki et al., 2020). Consequently, the search strategy employed in the WOSCC database was TS = (“*Akkermansia muciniphila*” OR “*A. muciniphila*”). The time span is from January 2004 to December 2023. Following a rigorous three-round screening process, we narrowed this down to 1,746 documents eligible for further analysis (Figure 1A). The search strategy utilized in the Innography database was @ (abstract, claims, title) (“*Akkermansia muciniphila*” OR “*A. muciniphila*”). A total of 1,160 *A. muciniphila* Patents were collected. After exclusion of 449 expired patents, 661 active patents (222 grants, 439 applications) were gone on with analysis and visualization (Figure 1B). Figure 1 provides a detailed overview of the extraction time, screening process, and analyzed metrics in both databases.

To ensure the accuracy and completeness of our keyword selection, we performed keyword cleaning, which involved merging synonyms, aliases, and singular and plural forms. This process was conducted independently by both authors and then mutually verified. Any disagreements or unresolved issues were resolved by the senior author, ensuring consistency in our keyword selection.

### 2.3 Bibliometric and visualization tools

CiteSpace, developed by Prof. Chaomei Chen from the School of Computing and Information at Drexel University, is a widely used tool in bibliometric research (Chen, 2006). This software offers effective and reliable text mining and knowledge visualization methods that enable researchers to explore research performance distributions, collaborations, current research status, emerging frontiers, and future trends.

In this study, CiteSpace was employed to investigate various aspects, including countries (time zone maps), institutions, keywords, reference parameters, and corresponding cluster visualization, timeline visualization, and burst detection. Additionally, R 4.3.1 Bibliometrix and Scimago Graphica were utilized to visualize the author distribution and country distribution of publications, respectively.

### 2.4 Evaluation

CiteSpace employs various evaluation metrics encompassing both structural metrics (such as betweenness centrality (BC), modularity (Q) score, and silhouette (S) score) and temporal metrics (burstiness).

BC is utilized to depict the extent of interaction among nodes in the network. Nodes with a BC value surpassing 0.1 are deemed central, possessing a commanding position over the majority of network resources (Wang et al., 2024b).

The Q score, ranging from 0 to 1, gages the density of connections within a module or community. A Q value surpassing 0.3 denotes a significant clustering structure (Wang et al., 2024b). As for the S score, which ranges from −1 to +1, it serves as a yardstick for assessing the strengths and weaknesses of clustering techniques employed. If the S score exceeds 0.7, the network is ascribed a high level of reliability (Wang et al., 2024b).

Burstiness refers to abrupt fluctuations, either a surge or decline, in the activity or frequency of elements during a specific time span (Sabé et al., 2023). Burstiness analysis proves valuable in identifying emerging dynamic concepts and potential research inquiries within a field. Moreover, it is particularly apt for examining sudden transformations or emerging trends in the advancement of a discipline, as well as visualizing previously active or pioneering nodes (Wang et al., 2024b).

Patent strength is an assessment of a patent’s vigor, determined by a variety of factors such as the number of patent claims, citations, legal cases or lawsuits, the length of the application, and the presence of related patents within the same family. Innography’s classification system divides patent strength into three categories: core, significant, and general patents. Core patents, positioned in the 80–100% range, are identified as the most impactful and innovative within their field. Significant patents, occupying the 30–80% range, represent considerable contributions but are not at the pinnacle of innovation. General patents, ranging from 0 to 30%, encompass a wider array of inventions with lower impact. The rating of patent strength not only serves to comprehensively depict the value of patent literature within a particular field but also facilitates a swift identification of research and development priorities.

### 2.5 Statistics

Descriptive statistics were employed for the analysis. Abstracts or full texts of publications and patents were reviewed as deemed appropriate.

## 3 Results

### 3.1 Academic research analysis

#### 3.1.1 Publication trends

**Figure 2:**
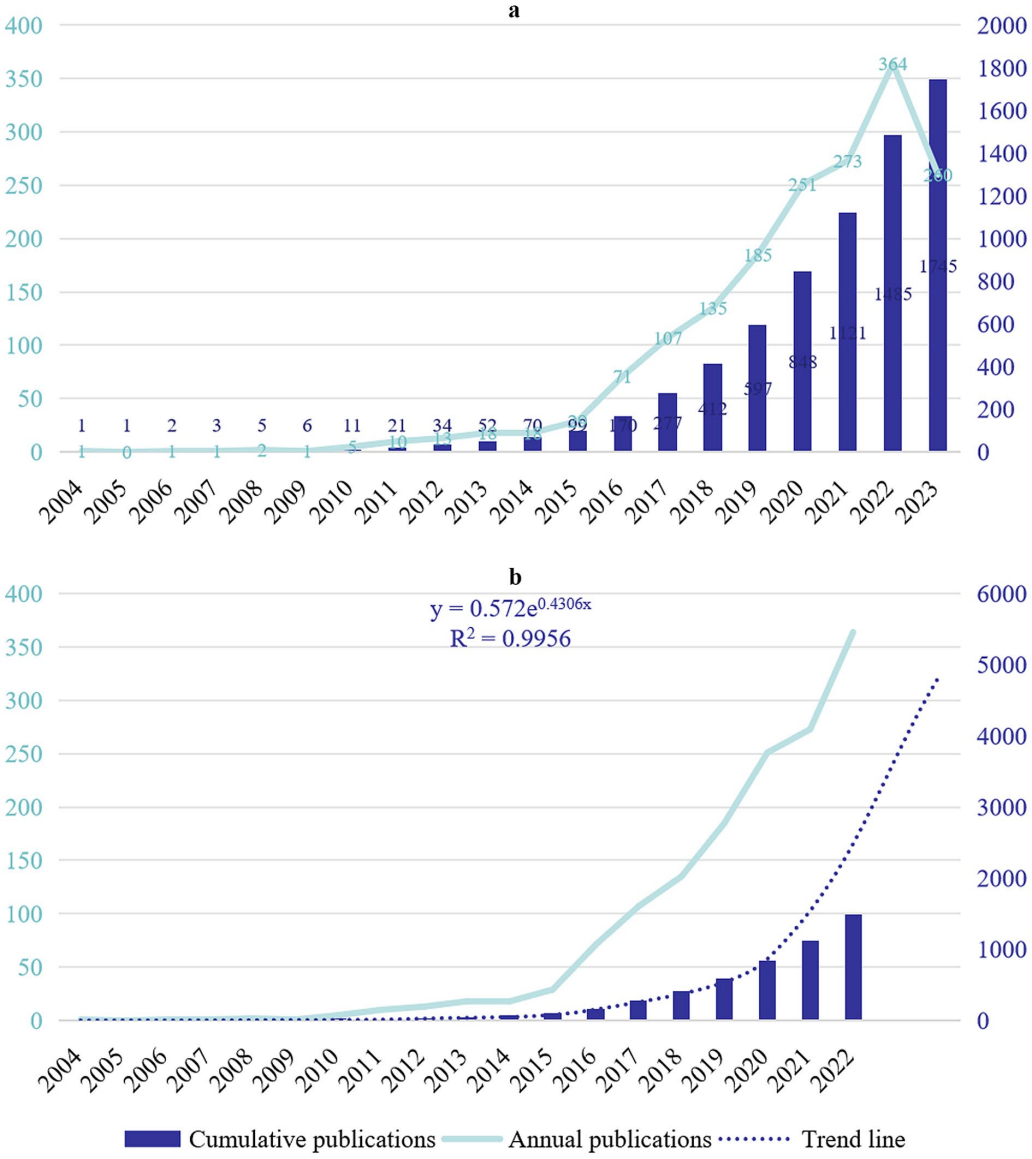
**(A)** Annual and cumulative outputs of publications. **(B)** Cumulative publications are calculated by excluding annual publications for the year 2023 and projecting backward two cycles.

The first research in the field emerged in 2004 and experienced a prolonged period of stagnation (Figure 2). It wasn’t until 2011 that the number of annual publications began to show some improvement, reaching a milestone of 10 and surpassing 100 in 2016. Subsequently, the field experienced rapid growth, with the publication count skyrocketing to 364 in 2022 (Figure 2A). Data from 2023 is not included from these calculations due to the incomplete yearly window (Figure 2). The trend line for cumulative publications (*y* = 0.572e^0.4306x^, *R*^2^ = 0.9956) demonstrates a remarkable exponential growth trajectory in the field. This trend suggests an estimated total of approximately 4,500 publications by 2024 (Figure 2B), highlighting the field’s remarkable research sustainability and scientific potential.

### 3.2 Countries

**Figure 3:**
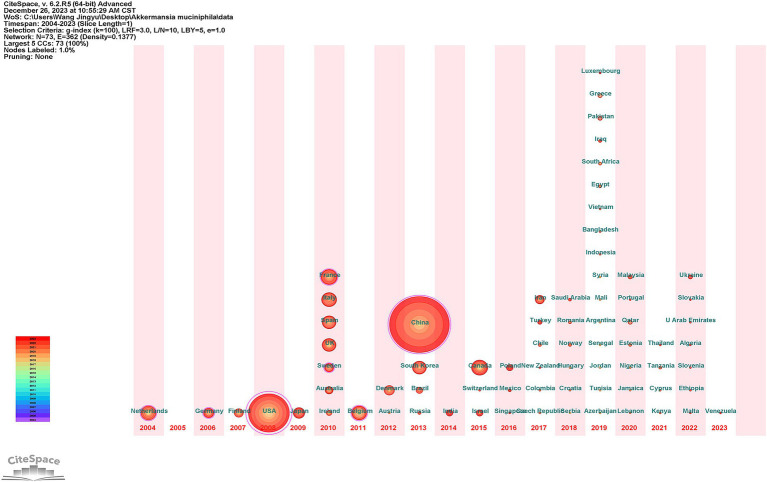
The CiteSpace-generated time zone map provides a visual representation of the earliest instances of *A. muciniphila* research conducted in different countries. The node size represents the number of publications. Nodes with rosy red outer edges are central nodes and their betweenness centrality (BC) is greater than 0.1; China (BC = 0.32), the United States (BC = 0.61), Sweden (BC = 0.18), France (BC = 0.17), Germany (BC = 0.16), Netherlands (BC = 0.14), and Belgium (BC = 0.11).

Research in this field has seen contributions from a total of 73 countries. Among them, there are seven core nodes, representing their important bridging role in the research network (Figure 3). The time zone map in Figure 3 identifies the Netherlands as the discovery site of *A. muciniphila*, aligning with the first published paper on the topic by a Dutch team in 2004 (Derrien et al., 2004). This early discovery likely catalyzed the initial wave of *A. muciniphila* research in Europe, with significant early contributions from countries like Germany and Finland (Figure 3). The year 2019 marks a pivotal point, witnessing the emergence of new research contributors such as Luxembourg, Greece, and Pakistan, signifying a shift to a more rapid phase of development in *A. muciniphila* research. This shift is further evidenced by a significant 44.4% annual growth in publications in the same year (Figure 2A).

**Figure 4:**
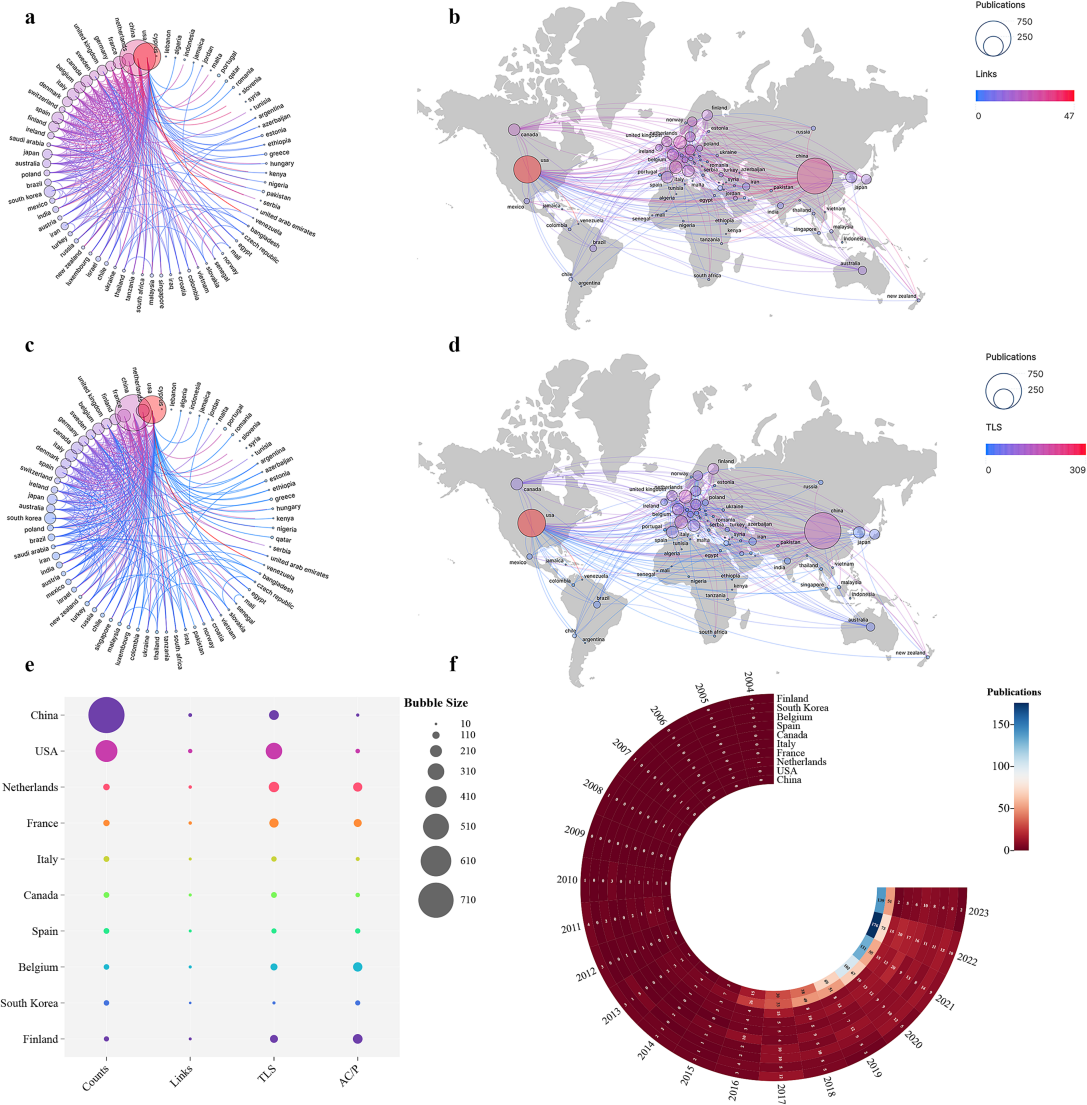
**(A–D)** The chord diagrams and map visualizations generated by Scimago Graphica seamlessly integrate links and TLS elements with the number of publications, providing a comprehensive visualization of the global distribution of research influence and collaborative endeavors. These visual representations offer a holistic and detailed portrayal of the interconnectedness and impact of research efforts worldwide. **(E)** Links, TLS, and AC/P for the top 10 high-productivity countries. **(F)** Annual fluctuations in academic output for the top 10 high-productivity countries.

Figure 4 illustrates the global collaboration network and its strength (as indicated by total link strength, TLS) in *A. muciniphila*-related research. In this figure, the size of each node corresponds to the number of publications from that country, while the line colors indicate the extent of the collaboration network (Figures 4A,B) and collaboration strength (Figures 4C,D). The United States (USA) emerges as a leader in publication volume, network breadth, and collaboration strength, particularly with countries depicted on the left side of Figures 4A,C. This dominance underscores the USA’s significant research capability and its international influence in this field. Notably, China, with the highest publication count, stands as the USA’s principal research partner, reflecting China’s rapidly growing research prowess in this domain. Mapping the data from Figures 4A,C onto a world map (as shown in Figures 4B,D) reveals distinct geographic research clusters, notably in Europe, North America (led by the USA), and East Asia (led by China). The collaboration among these regions is especially robust. However, some countries like Cyprus and Lebanon, as shown in Figures 4A,C, are yet to engage in significant cross-border or cross-regional research collaborations (Figures 4A,C).

Figure 4E and Table 1 provide additional insights, showcasing the links, TLS, and average citations per publication (AC/P) for the top 10 countries by publication volume. Notably, these leading countries are predominantly from East Asia, Europe, and North America, aligning with previous findings. This data underscores the remarkable research output, academic influence, and global collaboration spearheaded by Europe, the USA, and China in this field. Moreover, Figure 4F offers a visual representation of publication trends among these top 10 countries. Particularly striking is China’s ascent in *A. muciniphila* research, leading the world in publication volume since 2016. This prominence can likely be attributed to increased funding for *A. muciniphila* research in China and the country’s burgeoning pool of skilled researchers.

**Table 1:** Top 10 productive countries/regions in *A. muciniphila*, ranked by the number of publications.

Rank	Countries/regions	Counts	BC	Links	TLS	Citations	AC/P	Year
1	China	730	0.32	36	165	18,055	24.73	2013
2	United States	425	0.61	47	309	22,240	52.33	2008
3	Netherlands	94	0.14	28	178	14,241	151.50	2004
4	France	90	0.17	28	152	11,292	125.47	2010
5	Italy	79	0.03	21	69	3,195	40.44	2010
6	Canada	78	0.07	22	79	3,986	51.10	2015
7	Spain	78	0.06	19	65	5,296	67.90	2010
8	Belgium	74	0.11	22	105	11,466	154.95	2011
9	South Korea	72	0	10	22	4,551	63.21	2013
10	Finland	65	0.01	19	124	10,499	161.52	2007

### 3.3 Institutions

**Figure 5:**
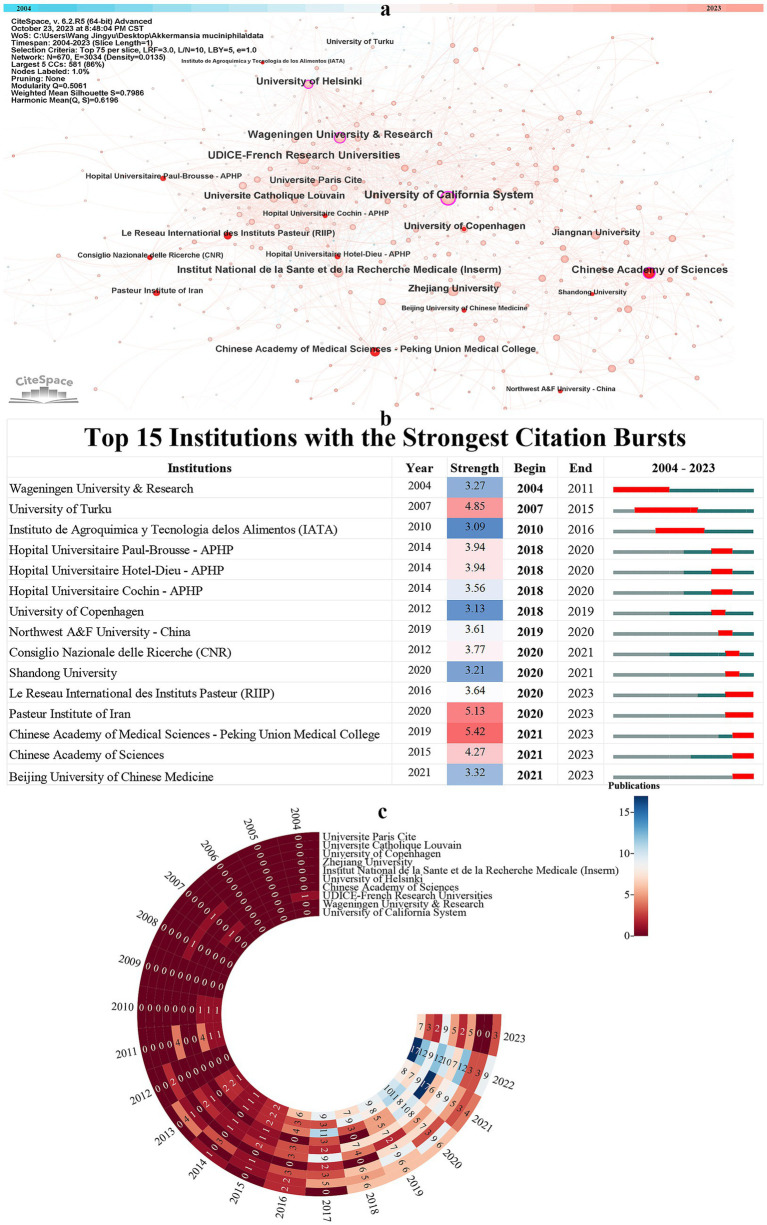
**(A)** The network visualization of academic institutions. **(B)** Top 15 institutions with the strongest citation bursts. **(C)** Annual fluctuations in academic output for the high-productivity institutions (top 10).

Figure 5A presents a global academic collaboration network encompassing 670 research institutions involved in *A. muciniphila* studies, as mapped out by CiteSpace. Prominent among these are the University of California system with 79 publications, the University and Research Institute of Wageningen with 63 publications, and the UDICE-French Research Universities, also contributing 63 publications (detailed in Table 2). This network visualization further highlights three pivotal bridging nodes, instrumental in fostering research connections: the University of California System (BC = 0.21), the Chinese Academy of Sciences (BC = 0.15), and Wageningen University & Research (BC = 0.13).

**Table 2:** Top 10 productive institutions in *A. muciniphila*, ranked by the number of publications.

Rank	Institutions	Counts	BC	H-index	AC/P	Year
1	University of California System	79	0.21	34	61	2010
3	Wageningen University and Research	63	0.13	35	185.29	2004
2	UDICE-French Research Universities	63	0.1	34	131.38	2010
4	Chinese Academy of Sciences	57	0.15	21	26.14	2015
6	University of Helsinki	51	0.1	33	190.43	2007
5	Institut National de la Sante et de la Recherche Medicale (Inserm)	51	0.06	29	180.27	2013
7	Zhejiang University	42	0.03	21	44.09	2017
8	University of Copenhagen	39	0.08	24	80.45	2012
9	Universite Catholique Louvain	38	0.02	28	264.21	2013
10	Universite Paris Cite	37	0.03	22	152.92	2014

Figure 5B enumerates the top 15 institutions identified based on burst intensity, a metric indicating rapid increases in publication activity. Notably, institutions with burst activities extending into 2023 are poised to become pivotal research centers, potentially driving major advancements in *A. muciniphila* research. Significantly, among these emerging research hubs, three are based in China. This underscores China’s rising prominence and potential for substantial contributions in the field of *A. muciniphila* studies.

In the last 7 years, there has been a notable surge in *A. muciniphila* research publications. This trend is evident in the top 10 high-productivity academic institutions, as shown in Figure 5C and Table 2. These leading institutions, hailing from Europe, North America, and East Asia, align with the geographical distribution identified in earlier analyses. The years 2020 and 2022 stand out as particularly prolific. Notably, the University of California system in the United States has been a key player in *A. muciniphila* research since 2010, peaking at 17 publications in 2022. Similarly, UDICE-French Research Universities began their contributions in 2010, culminating in 12 papers in 2022. Wageningen University, since its first paper in 2004, reached a high of 11 publications in 2017 and has maintained a steady output since. Remarkably, Institut national de la santé et de la recherche médicale (Inserm) published nearly 50 papers from 2020 to 2023, with 17 in 2021 and 12 in 2022 alone. Other prolific contributors include the University of Helsinki, Zhejiang University, University of Copenhagen, UC Louvain, Université Paris Cité, Chinese Academy of Medical Sciences-Peking Union Medical College, and Jiangnan University, further underscoring the global scope of *A. muciniphila* research (Figure 5C).

### 3.4 Author distribution

**Figure 6:**
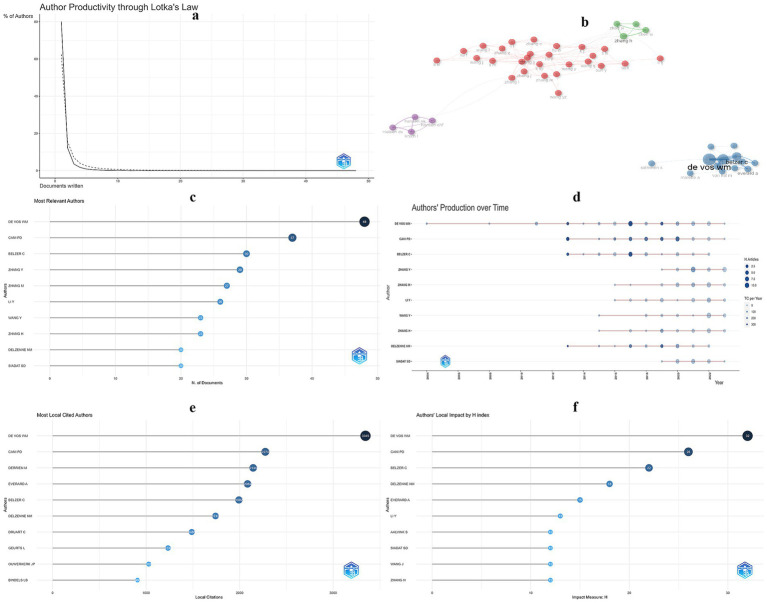
**(A)** Distribution of authors in the field of *A. muciniphila*. **(B)** Visualizing coupled clustering for the top 50 highly productive authors. **(C)** Top 10 authors ranked by number of publications. **(D)** Annual output of the Top 10 prolific authors. **(E)** Top 10 authors ranked by local citations. **(F)** Top 10 authors ranked by H index.

Figure 6A reveals a deviation from Lotka’s law in the *A. muciniphila* research field, particularly among authors with fewer than 10 publications. The Lotka’s law suggests that around 60% of authors have published a single paper, in the *A. muciniphila* field, this figure is closer to 80%, suggesting that very few authors are consistently and intensively involved in research. Further analysis shows that the top 50 high-productivity authors are organized into four distinct clusters (Figure 6B). Notably, cross-links between authors in three of these clusters suggest collaborative efforts. However, the blue cluster, led by Willem M de Vos, is comparatively more isolated. This isolation is notable, particularly as Willem M de Vos is identified as the most influential author in the field (Figures 6C–F). This pattern points to a diverse array of team structures among highly productive authors, with Willem M de Vos pursuing unique research trajectories.

Willem M de Vos stands as the foremost author in *A. muciniphila* research, with an impressive record of 48 publications, an H-index of 32, and 3,345 local citations (as depicted in Figures 6C–F). An analysis of the team’s 48 publications reveals three particularly influential papers, each exceeding 1,000 citations. The seminal 2004 paper titled “*Akkermansia Muciniphila* Gen. Nov., Sp. Nov., a Human Intestinal Mucin-Degrading Bacterium” has amassed 1,258 citations (Derrien et al., 2004). His team isolated the strain from human feces and named it in honor of Dr. Antoon D. L. Akkermans, a distinguished microbial ecologist, setting the stage for expansive research in this area. This was followed by a 2013 study, “Cross-talk between *Akkermansia muciniphila* and intestinal epithelium controls diet-induced obesity,” with 2,827 citations, highlighting *A. muciniphila*’s role in mitigating high-fat diet-induced weight gain and endotoxin levels in mice (Everard et al., 2013). Another notable publication in Nature Medicine in 2017, “A Purified Membrane Protein from *Akkermansia Muciniphila* or the Pasteurized Bacterium Improves Metabolism in Obese and Diabetic Mice,” with 1,117 citations, demonstrated how Amuc_1100, a protein from *A. muciniphila*, enhances the gut barrier and replicates the bacterium’s beneficial effects (Plovier et al., 2017). These contributions not only bolster the potential of *A. muciniphila* as a therapeutic agent against obesity and related disorders but also pave the way for its application as a next-generation probiotic.

### 3.5 Keywords

**Figure 7:**
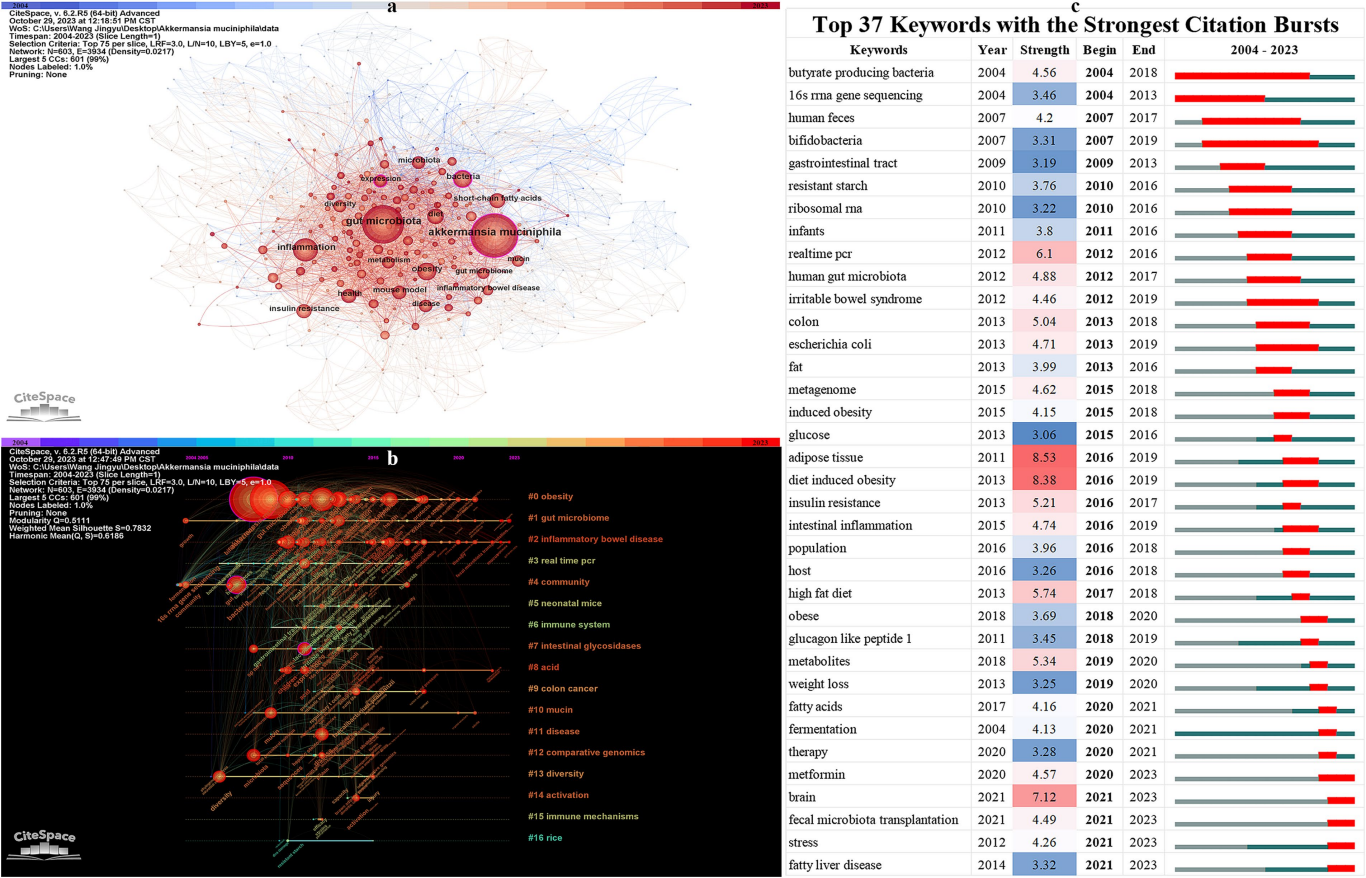
**(A)** Keyword network visualization. **(B)** Keyword timeline visualization. **(C)** Top 37 keywords with the strongest citation bursts.

In a thorough CiteSpace analysis, we extracted 603 key terms, interconnected by a total of 3,934 links, resulting in a densely populated visualization network (as seen in Figure 7A). Within this network, key terms appearing over 100 times were distinctly labeled. The three most prominent keywords were “*Akkermansia muciniphila*,” “gut microbiota,” and “inflammation” (Table 3). Furthermore, the network visualization spotlighted three central nodes, distinguished by their BC values: “expression” (BC = 0.15), “bacteria” (BC = 0.14), and “*Akkermansia muciniphila*” (BC = 0.13). These nodes represent pivotal concepts in *A. muciniphila* research, signifying key areas of focus and intersection within the field.

**Table 3:** The top 10 keywords with the highest frequency in *A. muciniphila*.

Rank	Keywords	Count	BC
1	*Akkermansia muciniphila*	1,196	0.13
2	Gut microbiota	965	0.04
3	Inflammation	336	0.05
4	Bacteria	229	0.14
5	Obesity	228	0.03
6	Short-chain fatty acids	176	0.02
7	Microbiota	159	0.03
8	Diet	155	0.03
9	Mouse model	154	0.05
10	Insulin resistance	150	0.04

Figure 7B’s timeline visualization provides a dynamic overview of evolving research trends in the field of *A. muciniphila*. It categorizes 603 keywords into 16 distinct clusters, demonstrating the diversity and breadth of research topics. The reliability and structure of these clusters are quantitatively validated by a modularity Q value of 0.511 and a silhouette S value of 0.7832, both of which suggest a high degree of clustering integrity and robustness. Notably, the three most prominent clusters are “#0 obesity,” “#1 gut microbiome,” and “#2 inflammatory bowel disease.” These clusters have maintained a consistent focus over time, highlighting sustained research interest in these areas.

Figure 7C’s burst test for keywords yielded 37 significant outcomes, effectively summarizing past research focal points, and indicating potential future directions in *A. muciniphila* studies. The evolution of this research can be categorized into three distinct phases. Initially, from 2004 to 2011, the focus was on the basic characterization of *A. muciniphila*, including its distribution, genomic structure, and function. Keywords from this era include “human feces,” “16S rRNA gene sequencing,” and “butyrate producing bacteria.” The subsequent period, 2012–2019, marked a shift toward investigating *A. muciniphila*’s abundance and its correlations with diseases, with a particular emphasis on its beneficial roles in metabolic and inflammatory conditions. Most recently, from 2020 to 2023, the keyword burst analysis has highlighted five emerging terms: “metformin,” “brain,” “fecal microbiota transplantation,” “stress,” and “fatty liver disease.” These terms represent the forefront of current research trends, signaling new directions and areas of investigation in the field of *A. muciniphila*.

### References

3.6

**Figure 8:**
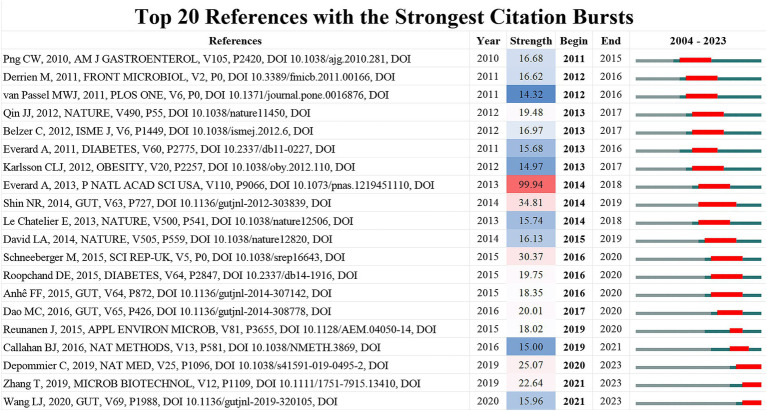
Top 20 references with the strongest citation bursts.

In *A. muciniphila* research, co-citation relationships are established when two or more publications are cited together within a single article. High co-citation counts indicate key foundational works in the field. Through citation burst analysis, which identifies references experiencing significant citation increases within a specific period, we have pinpointed the top 20 references with the strongest citation bursts, all published post-2010. Notably, three of these influential papers appeared in *Nature*, two in *Diabetes*, and four in *Gut*, as shown in Figure 8. Other significant publications were featured in *Science*, *Nature Medicine*, *Nature Methods*, *PNAS*, *ISME J*, *Obesity*, and *American Journal of Gastroenterology*. These studies predominantly focus on the link between *A. muciniphila* abundance and metabolic health. Many were published in high-impact journals, including *Nature* (3 papers), *PNAS* (1 paper), *Diabetes* (2 papers), and *Obesity* (1 paper).

Particularly between 2014 and 2015, *Gut* published three critical studies exploring the positive correlation between increased *A. muciniphila* populations and improved metabolic health in diabetic and obese adults. Among these top 20 papers, a 2013 *PNAS* study, with a strength score exceeding 99, markedly influenced the research direction in this field. This research highlighted the reduced abundance of *A. muciniphila* in obese and type 2 diabetic mice, and how its administration can counteract high-fat diet-induced metabolic disorders, including fat-mass gain, metabolic endotoxemia, adipose tissue inflammation, and insulin resistance (Everard et al., 2013). It revealed that *A. muciniphila* affects intestinal levels of endocannabinoids, which regulate inflammation, the gut barrier, and gut peptide secretion (Everard et al., 2013).

As research progresses, the therapeutic potential of *A. muciniphila* is increasingly being explored in immuno-therapy, neurological and hepatic diseases, and in combination with treatments like metformin. This comprehensive analysis thus provides valuable insights into the dynamic evolution, trends, and pivotal shifts in the *A. muciniphila* research landscape.

## 4 *Akkermansia muciniphila* patents visualization

### 4.1 Patent development trends

Analyzing the patent technology life cycle of *A. muciniphila* from 2004 to 2023 reveals its progression through three distinct stages—traditionally conceptualized as the embryonic, growth, and decline stages, omitting the mature stage in this specific case. During the embryonic stage (2004–2014), the volume of patent applications for *A. muciniphila* was limited, yet these applications exhibited a high grant rate. This pattern suggests significant potential for innovation at these early stages. The growth stage, spanning 2015 to 2020, marked a period of rapid development in *A. muciniphila* patent technology. The number of applications increased markedly, from approximately 50–200 annually. However, the grant rate during this period was comparatively low, around 20%, indicating that despite escalated investment in R&D, the rate of successful patent conversions was modest, highlighting areas for potential enhancement. The decline in patent applications and grants from 2021 to the present (Supplementary Figure S1) is most likely due to the lag in patent applications. Thus, this does not mean that the translational potential of the field has diminished in recent years.

### 4.2 Patent source jurisdictions

Supplementary Figures S2A–C delineate the distribution of patents related to *A. muciniphila*, categorizing them into total patents (1,160), active patents (661), and granted patents (222) across various jurisdictions. These figures highlight the United States, Europe, and China as leading contributors in the *A. muciniphila* patent arena, dominating in terms of patent applications, active patents, and grants. Notably, the USA emerges as the most prominent target market in the global context of *A. muciniphila* patents. Additionally, international organizations, namely the World Intellectual Property Organization (WIPO) and the European Patent Office (EPO), play a crucial role in this domain. Their efforts in promoting and protecting *A. muciniphila*-related patents are essential for nurturing innovation and maintaining intellectual property rights in this rapidly evolving research field.

### 4.3 *Akkermansia muciniphila* patents clustering

**Figure 9:**
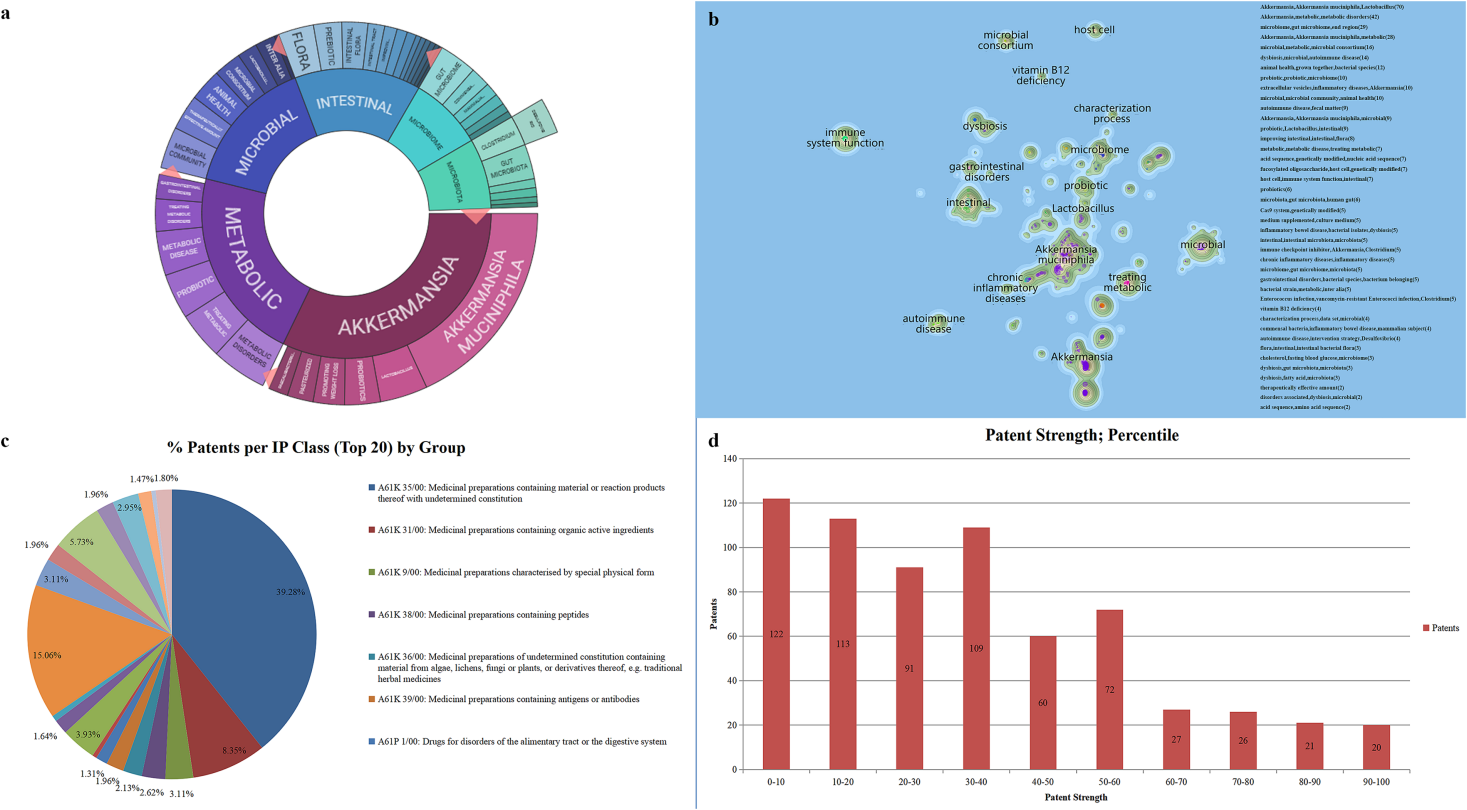
**(A)** Patent text clustering. **(B)** Patented landscape visualization. **(C)** Top 20 intellectual property categories and corresponding percentages. **(D)** Patent strength distribution of 661 active patents.

Innography text clustering is a method employed to quickly distill the key technological themes within a specific domain. It achieves this by extracting and grouping recurring keywords from patent titles and abstracts. This approach provides a broad overview of the field’s distribution, highlighting subfields, nascent technologies, and general trends in technological development. Figure 9A showcases a text clustering diagram for 661 active *A. muciniphila* patents. Within this diagram, the inner ring highlights the primary technological themes, while the outer ring identifies secondary themes. The segments’ sizes in this pie chart correlate with the number of patents in each category. Further refining this analysis, Figure 9B, derived from Figure 9A, presents a patent landscape map offering a comprehensive view of *A. muciniphila* patent distribution. Specifically, in the primary technical fields related to *A. muciniphila*, the categories of “Metabolic,” “Microbial,” and “Intestinal” dominate, comprising over 70% of the patent applications (as indicated in Figure 9A). This underscores *A. muciniphila*’s significant potential in managing metabolic diseases, microbial regulation, and gut health. Additionally, as an emergent probiotic, *A. muciniphila* is linked to 70 patents concerning the first-generation probiotic “*Lactobacillus*” and “chronic inflammatory diseases,” showcasing its considerable promise for future research and application in these areas.

### 4.4 International patent classification of *Akkermansia muciniphila* patents

The international patent classification (IPC) system, a universally recognized framework for classifying patent documents, offers vital insights into the technical orientation of patents in specific fields. An analysis of the IPC classifications for *A. muciniphila*-related patents leads to three key observations (Figure 9C):

- **Medical field dominance**: The majority of *A. muciniphila* patents are centered in the medical sector. Among the top 20 IPC subclasses for these patents, approximately 75% (15 subclasses) are related to medical applications, underscoring the field’s predominant focus.
- **Potential in disease treatment**: *A. muciniphila*-related patents encompass a broad spectrum of diseases, indicating its versatile therapeutic potential. These patents address various conditions including cancer, neurological disorders, and metabolic diseases, highlighting the breadth of its applicational scope.
- **Diverse application methods**: The functionality of *A. muciniphila* extends across multiple forms of applications. This includes its use in pharmaceuticals, diagnostic reagents, and health supplements.

These findings collectively illustrate the significant presence of *A. muciniphila* in medical research and its expansive utility in treating a wide range of diseases through varied application strategies.

### 4.5 *Akkermansia muciniphila* patents strength

Patent strength serves as a critical metric in evaluating a patent’s potential value, with higher strength indicating greater technical sophistication, novelty, and breadth of protection. As depicted in Figure 9D, a substantial portion, over 93% (620 out of 661 patents), falls below the 80% threshold and only 41 patents qualify as high-strength. This suggests that the bulk of *A. muciniphila* patents are of lower quality and impact, reflecting a less mature state in terms of technical development and influence, indicating a need for increased investment in research and development to enhance the quality and impact of future patents in this area.

## 5 Discussion

### 5.1 The trajectory of *Akkermansia muciniphila* research

Following its initial isolation from a healthy individual at the Laboratory of Microbiology in Wageningen, *A. muciniphila* research experienced a decade of gradual development (Derrien et al., 2004). This period saw advancements in analytical tools, optimization of isolation and cultivation methods, and the acquisition of its genomic information (van Passel et al., 2011). As research teams diversified, exploratory work in animal disease models began to garner attention in *A. muciniphila* studies. Observational studies revealed a notable negative association between *A. muciniphila* abundance and obesity/diabetic conditions (Crovesy et al., 2020; Everard et al., 2011; Everard et al., 2014; Macchione et al., 2019) in both mice and humans, sparking interest in its potential therapeutic role for obesity. A pivotal 2013 study, a collaboration between UCLouvain (Belgium) and UWageningen (Netherlands), demonstrated that daily administration of live *A. muciniphila* in mice could reverse HFD-induced metabolic disorders, including fat-mass gain, metabolic endotoxemia, adipose tissue inflammation, and insulin resistance (Everard et al., 2013). This breakthrough quickly translated into human trials; the 2015 “Microbes4U” study validated the safety and tolerability of ingesting either live or pasteurized *A. muciniphila* in 40 healthy adults. Current understanding of *A. muciniphila*’s mechanism in obesity prevention and treatment includes its role in promoting mucus layer renewal in the intestine, aiding in maintaining the intestinal barrier, enhancing energy loss in feces, and reducing metabolic endotoxin absorption (Everard et al., 2011; Aggarwal et al., 2022; Depommier et al., 2020; Qu et al., 2021; Shin et al., 2014). These findings position *A. muciniphila* as a potential key player in the development of obesity-related diseases (Dao et al., 2016; van der Lugt et al., 2019; Yoon et al., 2021). Overall, the accumulated research offers promising prospects for future therapeutic or preventive applications of *A. muciniphila* in individuals with pre-obesity or obesity.

*A. muciniphila*’s role extends beyond metabolic diseases, encompassing significant implications for immune-related conditions, including type 1 diabetes (Hänninen et al., 2018; Hansen et al., 2012; Routy et al., 2018). Hansen et al. in 2012 first illuminated a potential link between *A. muciniphila* and type 1 diabetes onset (Hansen et al., 2012). Their findings indicated that newborn and adult NOD mice treated with vancomycin exhibited a reduced incidence and delayed onset of type 1 diabetes, correlating with an over 85% abundance of *A. muciniphila* (iMSMS Consortium, 2022), suggesting a possible protective effect (Hansen et al., 2012). Additionally, *A. muciniphila*’s beneficial impact extends to intestinal inflammation. Notably, it has shown promise in mitigating colitis, with AmEVs (*A. muciniphila*-derived extracellular vesicles) protecting against dextran sulfate sodium (DSS)-induced colitis in mice (Png et al., 2010; Kang et al., 2013). Subsequent studies have observed *A. muciniphila*’s effectiveness in restoring gut barrier function and ameliorating DSS-induced colitis, evidenced by reduced weight loss, colon length shortening, and lower histopathology scores (Bian et al., 2019; Zhai et al., 2019). Despite some contradictory results, these studies collectively position *A. muciniphila* as an emerging protective agent against inflammatory diseases (Png et al., 2010; Kang et al., 2013; Zhai et al., 2019; Ganesh et al., 2013; Seregin et al., 2017). Recent research has also highlighted the gut microbiome, including *A. muciniphila*, as a marker and potential enhancer of clinical responses to immune checkpoint inhibitor (ICI) treatments (Routy et al., 2018; Routy et al., 2018). Routy et al.’s study on patients with non-small cell lung cancer and renal cell carcinoma receiving PD-1-based immunotherapy revealed a significant enrichment of *A. muciniphila* in the microbiota of responders (Hänninen et al., 2018; Routy et al., 2018); In non-responding patients, treatment with *A. muciniphila* strain *Akk*p2261 could restore ICI response (Derosa et al., 2022), underlining its potential role in augmenting immunotherapy effectiveness. Similarly, co-treatment with *A. muciniphila* strengthened IL-2 antitumor effects in certain cancer models (Shi et al., 2020). In prostate cancer, *A. muciniphila* showed a positive correlation with response to androgen deprivation therapy, and in animal models, it improved the therapy’s efficacy (Daisley et al., 2020; Terrisse et al., 2022). However, human clinical trials are essential to validate these findings further.

**Figure 10:**
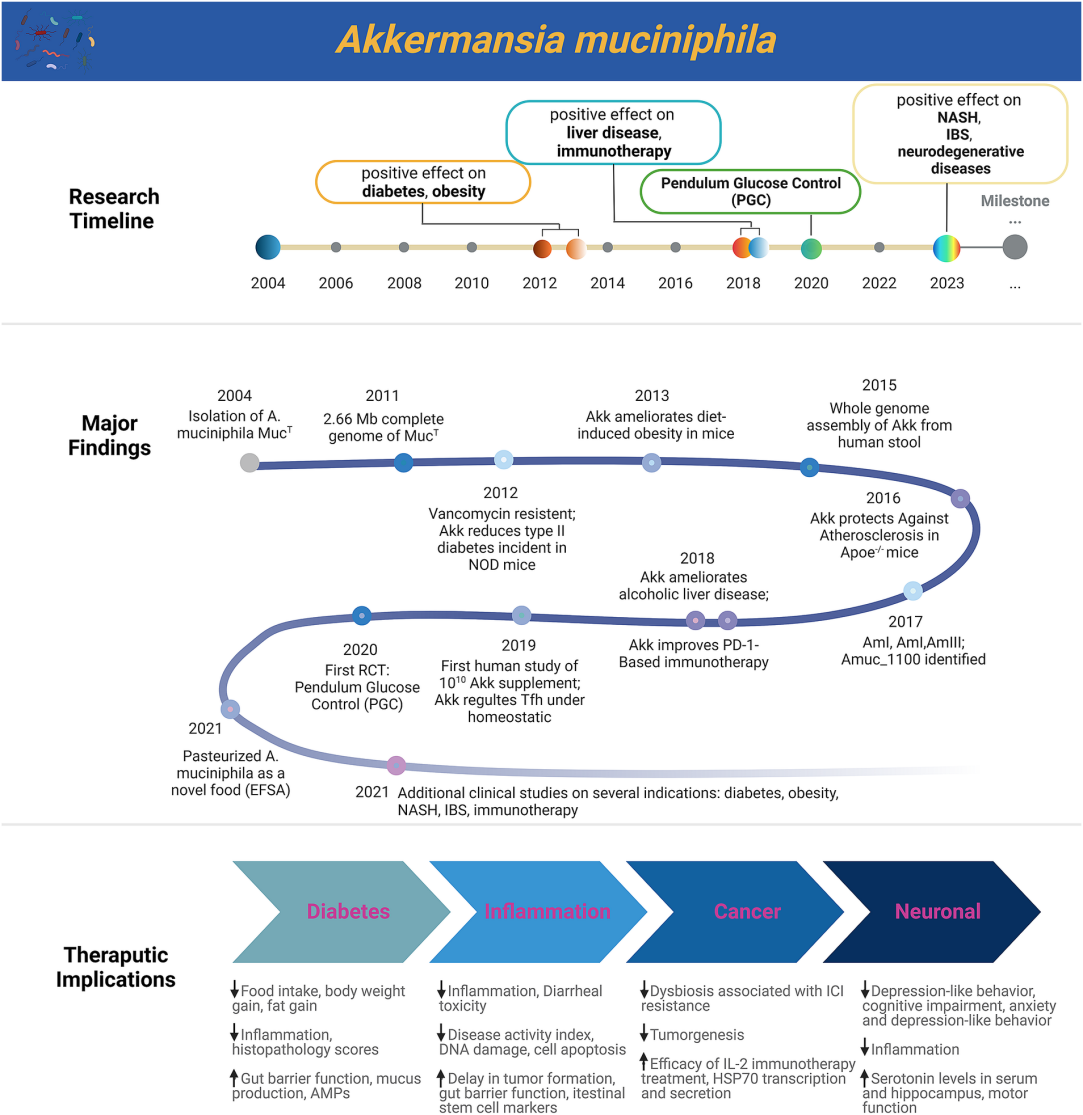
A timeline of major advances related to *A. muciniphila* is displayed. NASH, nonalcoholic steatohepatitis; IBS, irritable bowel syndrome. Created with BioRender.com.

In recent years, an association between *A. muciniphila* and neurodegenerative diseases such as autism and amyotrophic lateral sclerosis (ALS) has been observed (Blacher et al., 2019; Liu et al., 2019; Nitschke et al., 2020). Contrary to the above-mentioned diseases where *A. muciniphila* is depleted, it is more abundant in the microbiota of patients with neurological disorders or stroke (Li et al., 2019; Nishiwaki et al., 2020; iMSMS Consortium, 2022). Although confounding factors, such as medication and transit time reduction, may complicate these findings, there is no direct evidence supporting a role of *A. muciniphila* in promoting progression or symptoms of these diseases (Cani et al., 2022). On the other hand, intervention studies suggest that increased *A. muciniphila* levels may have a protective effect in multiple sclerosis (MS)(Liu et al., 2019) and ALS (Gotkine et al., 2020). Although research in these areas remains limited, further exploration is needed. As a promising next-generation probiotic, *A. muciniphila* continues to attract research interest, particularly in these emerging areas of study, as depicted in Figure 10.

### 5.2 The parallel progression of academic findings and patent trends

Advancements in metagenomics, culturomics, big data, artificial intelligence, and systems biology have catalyzed *A. muciniphila* research into a phase of clinical translation. Groundbreaking discoveries in basic research are progressively leading to marketable clinical applications, with patent trends reflecting similar developments. *A. muciniphila* research has evolved through three primary phases:

**Foundational phase (2004–2011)**: Focused on the strain’s basic properties, like distribution, genomics, and functionality. Despite fewer patent applications, a high grant rate indicated significant innovation potential.

**Expansion phase (2012–2019)**: Research shifted to exploring *A. muciniphila*’s relationship with diseases, particularly metabolic and inflammatory disorders. This phase marked increased interest from pharmaceutical and biotech industries, foreseeing a potential market for *A. muciniphila*-related products and technologies.

**Growth and diversification phase (2015–2020)**: Following the 2013 UCLouvain and UWageningen study demonstrating *A. muciniphila*’s benefits in reversing metabolic disorders in mice, and the subsequent “Microbes4U” study in 2015, patent technology entered rapid development. This period saw expanded research into broader health aspects, including non-alcoholic fatty liver disease, stress, neurology, and Fecal Microbiota Transplantation (FMT) therapies.

June 2020 marked a milestone with Pendulum Therapeutics launching Pendulum Glucose Control (PGC), the first *A. muciniphila*-containing food product, clinically proven safe (NCT04228003). This spurred global patent applications and product development in various *A. muciniphila* applications. However, recent years have witnessed a decline in patent numbers, possibly due to the COVID-19 pandemic’s delayed effects or reaching a bottleneck in basic research, highlighting the need for breakthrough technological advancements.

### 5.3 Global pattern of *Akkermansia muciniphila*’s commercialization

The United States and Belgium hold leading positions in *A. muciniphila* patent activity, with significant contributions also from China and other countries. This global distribution of patents, facilitated by organizations such as WIPO and EPO, underlines the international significance and collaborative efforts in *A. muciniphila* research and development. The involvement of these key entities not only fosters and safeguards patents but also underscores the importance of international cooperation in expanding *A. muciniphila* applications across various industries. There is a notable parallel between the geographic distribution of *A. muciniphila* patents and academic publications, indicating that regions with stronger academic foundations tend to be more successful in translating research findings into commercial products. This trend is predominantly observed in developed countries, which often lead the top 10 list in terms of research productivity. These countries benefit from robust academic environments, facilitating the seamless transition from research to market applications. Moreover, factors such as governmental policy support, market demand, and the overall innovation ecosystem are critical in directing the course of *A. muciniphila* research and its commercial exploitation. Additionally, the variation in *A. muciniphila* distribution among different ethnic groups and regions also influences the academic and commercial landscape. For instance, a study analyzing over 2,000 Akkermansia genomes revealed that *A. muciniphila*, predominantly found in Western and Chinese populations, is the dominant species with high genomic similarity (Karcher et al., 2021). In contrast, research comparing the gut microbiome of non-industrialized populations, such as the Hadza tribe, with those in industrialized settings showed a reduced abundance of certain microbiota, including *A. muciniphila* (Fragiadakis et al., 2019; Smits et al., 2017). These differences raise questions about whether such variations are attributable to methodological factors like sample preservation and DNA extraction, or if they reflect true biological diversity. Thus, these multifaceted factors - encompassing economic, academic, ethnic, and methodological aspects - collectively shape the current global pattern of *A. muciniphila* research competition and its commercialization trajectory (Supplementary Figure S2).

### 5.4 Key barriers to a broader application

**Figure 11:**
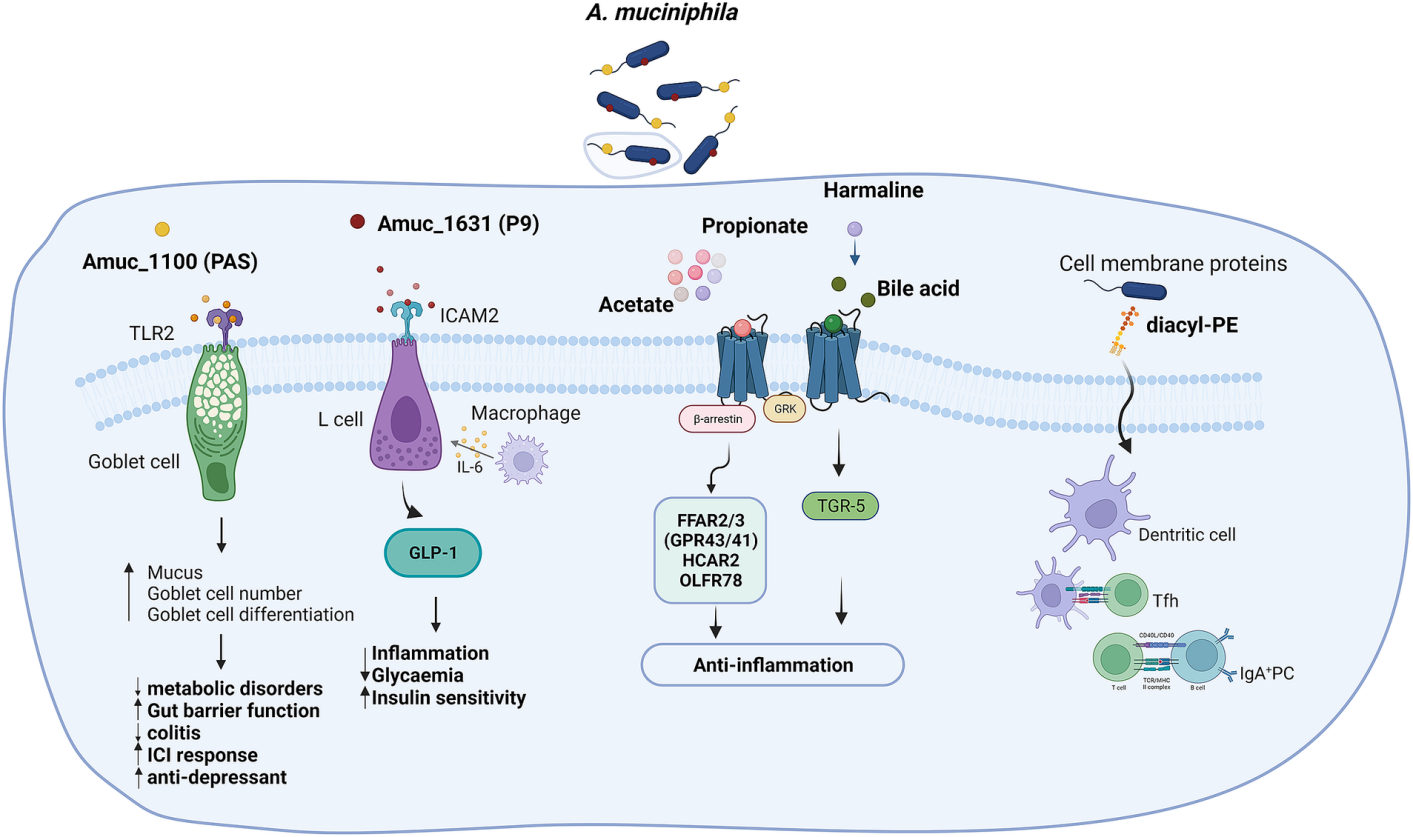
Known mechanisms underlying *A. muciniphila*’s beneficial effects on human health. TLR2, Toll like receptor 2; ICAM2, Intercellular adhesion molecule 2; TGR-5, Takeda G protein-coupled receptor 5; Tfh, T follicular helper cells, GRK, G protein-coupled receptor kinase. Created with BioRender.com.

As a promising next-generation probiotic, *A. muciniphila* holds potential as a therapeutic target for various diseases. However, several challenges impede its research and application:

- **Isolation and identification:** The isolation and identification of *A. muciniphila* are complex processes, highly dependent on specific culture conditions. While it is divided into four phylotypes, research mainly focuses on the Muc^T^ strain. Variability among human-associated *A. muciniphila* strains, reported in preclinical trials, requires further investigation to understand their consistency and reproducibility (Becken et al., 2021; Yang et al., 2020).
- **Genomic and antibiotic resistance:** Issues with genome editability and multiple antibiotic resistances pose additional hurdles for industrial applications.
- **Mechanism understanding:** Current research predominantly explores *A. muciniphila*’s disease associations, with incomplete knowledge about its interaction mechanisms with the host. While known factors like the PAS protein Amuc_1100 (Plovier et al., 2017; Ottman et al., 2017), Glucagon-like peptide-1-inducing protein P9 Amuc_1631 (Yoon et al., 2021), diacyl-PE in the outer membrane (Bae et al., 2022), and several typical metabolites such as acetate (Derrien et al., 2004), propionate (Zarrinpar et al., 2014), and harmaline (Xie et al., 2023) have been identified, research on the immune-modulating effects of *A. muciniphila* is still evolving and mechanisms remain poorly understood (Figure 11).
- **Safety and efficacy in humans:** Direct intervention of *A. muciniphila* is mainly carried out in animal models, and the translational applicability of results from animal models to humans, especially considering the differences in intestinal probiotic effects, remains a critical area for attention. Preliminary data from “Microbes4U” showed that people have no adverse reactions to oral administration of *A. muciniphila* in the short term, but its long-term effects and whether it produces toxic side effects need to be further verified in more human clinical trials.
- **Dose-dependence:** Some studies suggest that *A. muciniphila*’s efficacy is dose-dependent, and lower doses may not yield effective results.
- **Contradictory findings:** Despite its potential in host health regulation and disease treatment, contradictory findings in some studies caution against indiscriminate use. Research by Chassaing et al. and Seregin et al. has indicated potential adverse effects, such as increased intestinal permeability and severity of colitis under certain conditions (Seregin et al., 2017; Chassaing et al., 2015).
- **Pathogenicity in susceptible populations:** The possibility of *A. muciniphila* causing mucosal immune abnormalities or pathogenicity in genetically susceptible populations, or its interaction with other bacteria, raises concerns that need addressing in future research.

These limitations underscore the need for further investigation into *A. muciniphila*’s properties, ensuring safe and effective clinical applications. The current research trajectory also points to the importance of understanding its effects across diverse populations and conditions.

### 5.5 Challenges

High-strength patents (over 80%) in the *A. muciniphila* domain are predominantly concentrated in medical and biotechnological applications. These applications include treatments for metabolic disorders, cancer, and immune system modulation, utilizing live *A. muciniphila*, specific *Akkermansia* sp. strains, microbial consortia, or therapeutic polypeptides. It is crucial to view *A. muciniphila* Muc^T^ not as a standalone treatment, but as a complementary adjuvant, potentially augmenting individual health and delaying disorder progression when combined with other lifestyle interventions. However, the progress made with the Muc^T^ strain, including its approval as a novel food ingredient, has yet to be replicated with other strains. The commercialization of new live bacterial drugs, including *A. muciniphila*-based therapies, is currently in a nascent and ambiguous state. Their development and market introduction are impeded by the need for extensive basic research and clinical trials, coupled with limited approved indications and a general lack of robust evidence. To address these challenges, further exploration into the molecular mechanisms of *A. muciniphila*’s interaction with the host is essential. This includes investigating the potential production of toxic metabolites and identifying any virulence factors through genomics, metabolomics, and other multi-omics approaches. The patent technology development for *A. muciniphila* has witnessed a phase of rapid growth, now followed by a decline. To foster sustainable progress, there is a need for breakthroughs in basic research and enhancement of the actual patent conversion rate. Improving patent transfer mechanisms and bolstering the practical application value of patents are also critical. Moreover, strengthening international cooperation can facilitate technology exchange and spur innovation (Table 4).

**Table 4:** Currently recruiting clinical trials of *A. muciniphila*.

NCT number	Study status	Conditions	Enrollment	Sex	Age	Interventions	Phases	Locations
NCT05738746	Recruiting	Stress|Healthy|Dietary supplement	200	ALL	Adult, Older_Adult	Dietary_Supplement: Pasteurized *Akkermansia muciniphila*|Dietary_Supplement: Placebo	NA	Poland
NCT05865730	Recruiting	Carcinoma, Renal cell|Carcinoma, Non-small-cell lung	122	ALL	Adult, Older_Adult	Other: Live bacterial product - *Akkermansia muciniphila*	Phase2	France
NCT05720299	Recruiting	Obesity associated disorder	120	ALL	Adult, Older_Adult	Drug: Treatment with *Akkermansia muciniphila*|Other: Treatment with placebo	NA	China
NCT05795972	Recruiting	Diabetes	60	ALL	Adult, Older_Adult	Dietary_Supplement: inulin|Other: standard therapy	NA	Italy
NCT05022524	Recruiting	Depression, anxiety	10	ALL	Child, Adult	Dietary_Supplement: Acetate (apple cider vinegar)	Phase1	Canada
NCT05762887	Recruiting	Bipolar disorder	84	ALL	Adult, Older_Adult	Other: Probiotic group|Other: Placebo group	NA	Brazil
NCT05303493	Recruiting	NSCLC Stage IV|Melanoma Stage IV|Unresectable Melanoma|Advanced non-small cell lung cancer	45	ALL	Adult, Older_Adult	Biological: Camu Camu Capsules (Camu Camu powder encapsulated) (500 mg each) + ICI	Phase1	Canada
NCT04924374	Recruiting	Lung cancer	20	ALL	Adult, Older_Adult	Dietary_Supplement: Microbiota transplant plus anti PD1 therapy|Drug: anti PD1 therapy	NA	Spain

As *A. muciniphila* research and application are still in early stages, focusing on key areas is vital for future progress:

- Elucidating the molecular mechanisms of *A. muciniphila*’s interaction with the host, identifying crucial target molecules and pathways.
- Conducting comprehensive human clinical trials to evaluate its safety, efficacy, and tolerability.
- Investigating potential toxicity and developing safe, effective *A. muciniphila*-based preparations.

Addressing these areas can propel *A. muciniphila* toward becoming a safe, effective therapeutic or preventive agent for various diseases.

## 6 Conclusion

This is the first comprehensive analysis employing bibliometric analysis and visualization using both the WOSCC and Innography databases. Our study not only reveals the global research landscape and commercial distribution in the field of *A. muciniphila* but also delves into the academic frontiers and commercial trends within this domain. *A. muciniphila* presents a horizon of opportunities in health and disease management. To harness its full potential, future research must focus on elucidating its molecular interactions with the host, conducting rigorous human trials, and developing effective, safe formulations. Addressing these challenges will be crucial for *A. muciniphila* to transition from a scientific curiosity to a mainstay in therapeutic and preventive healthcare.

## Data Availability

The original contributions presented in the study are included in the article/Supplementary material, further inquiries can be directed to the corresponding author.
