# Supplementary material for: The worldview of Akkermansia muciniphila, a bibliometric analysis
Source: Front Microbiol. 2025 Mar 4;16:1500893. doi: 10.3389/fmicb.2025.1500893 (PMC11913835; doi:10.3389/fmicb.2025.1500893)
Supplement: Supplementary file 1 [file Data_Sheet_1.zip › Supplementary Figures/Supplementary Figures Legends.docx]

**Supplementary Figure 1.** a. Number of patent applications, number of active patents, number of granted patents, and patent grant rate in the field of *A. muciniphila*, presented in a nature-inspired style, year by year. b. Top 4 application sources for 1160 patents. c. Top 4 application sources for 661 active patents. d. Top4 application sources for 222 granted patents.

**Supplementary Figure 2.** a. Total number of 1160 patents by source jurisdiction. b. 661 active patents by source jurisdiction. c. 222 granted patents by source jurisdiction.
