# Supplementary figures and images for: The worldview of Akkermansia muciniphila, a bibliometric analysis
Source: Front Microbiol. 2025 Mar 4;16:1500893. doi: 10.3389/fmicb.2025.1500893 (PMC11913835; doi:10.3389/fmicb.2025.1500893)

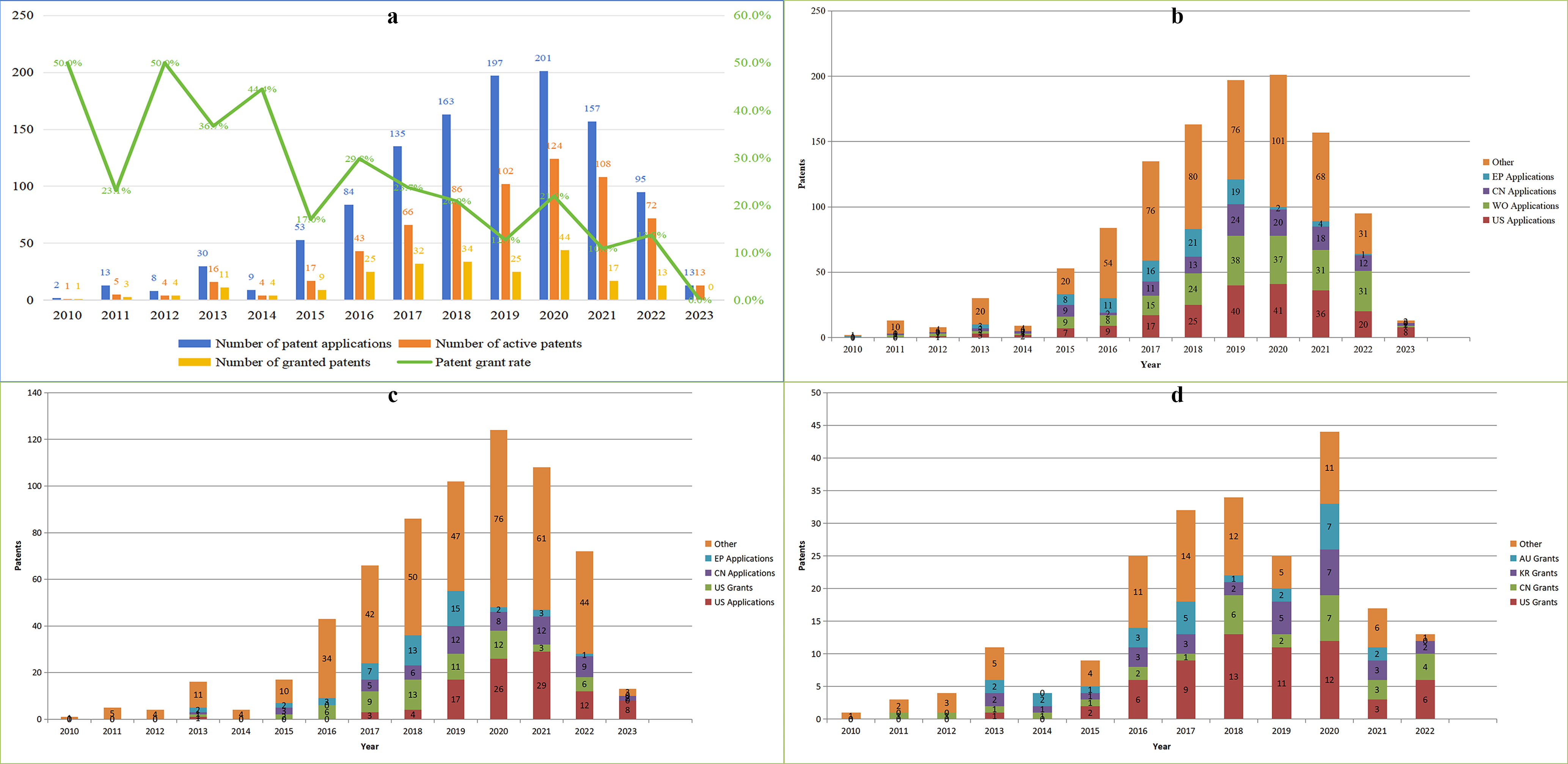

Supplement: Supplementary file 1 [file Data_Sheet_1.zip › Supplementary Figures/Supplementary Fig 1.tif]

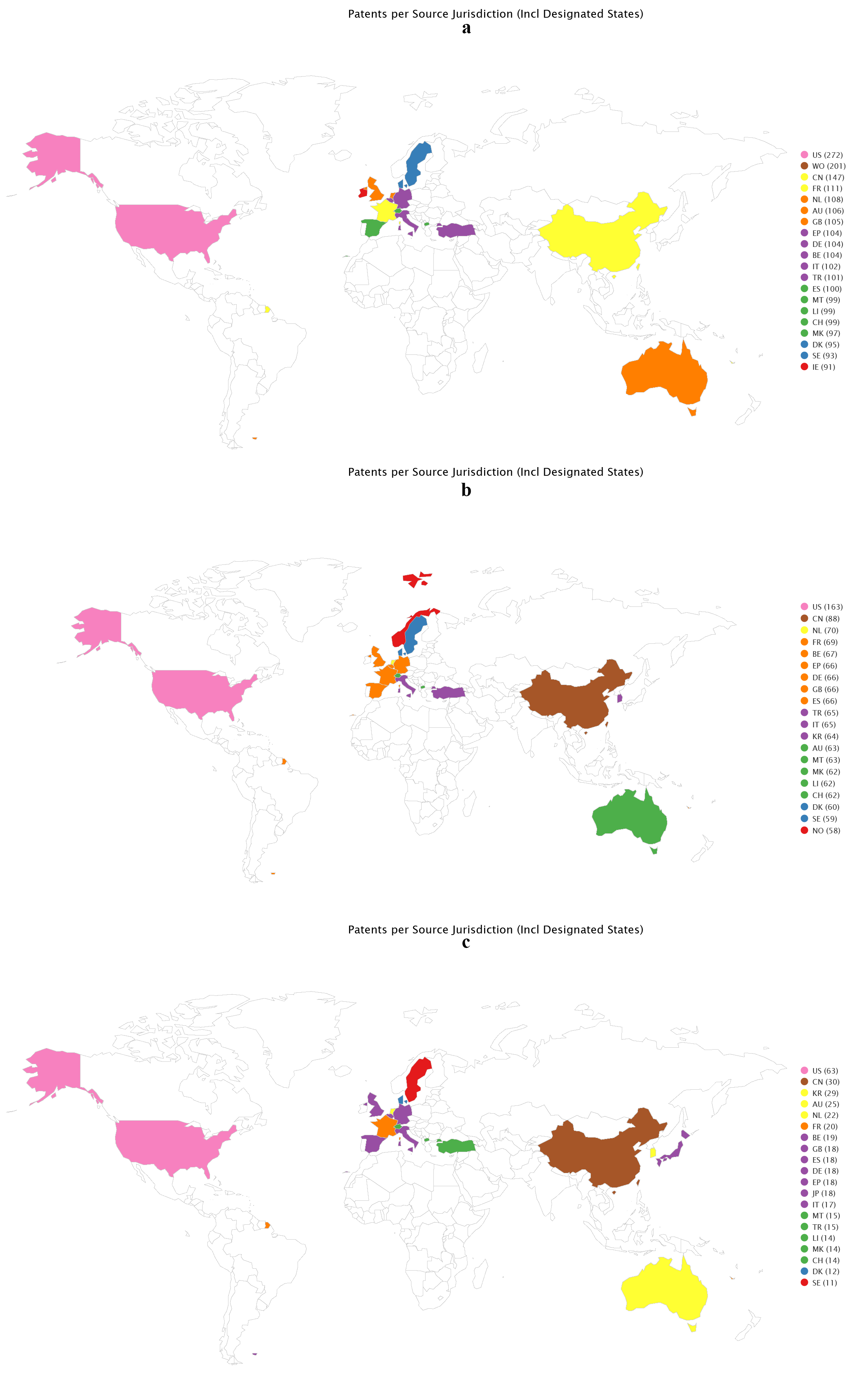

Supplement: Supplementary file 1 [file Data_Sheet_1.zip › Supplementary Figures/Supplementary Fig 2.tif]
