# Supplementary material for: The worldview of Akkermansia muciniphila, a bibliometric analysis
Source: Front Microbiol. 2025 Mar 4;16:1500893. doi: 10.3389/fmicb.2025.1500893 (PMC11913835; doi:10.3389/fmicb.2025.1500893)
Supplement: Supplementary file 2 [file Table_1.docx]

**Supplementary Table 1. 48 publications published by Willem M de Vos' team. TC: total citations.**

| **Year** | **Title** | **Journal** | **DOI** | **TC** |
| --- | --- | --- | --- | --- |
| 2004 | AKKERMANSIA MUCINIPHILA GEN. NOV., SP NOV., A HUMAN INTESTINAL MUCIN-DEGRADING BACTERIUM | INTERNATIONAL JOURNAL OF SYSTEMATIC AND EVOLUTIONARY MICROBIOLOGY | 10.1099/ijs.0.02873-0 | 1258 |
| 2008 | THE MUCIN DEGRADER AKKERMANSIA MUCINIPHILA IS AN ABUNDANT RESIDENT OF THE HUMAN INTESTINAL TRACT | APPLIED AND ENVIRONMENTAL MICROBIOLOGY | 10.1128/AEM.01226-07 | 435 |
| 2011 | MODULATION OF MUCOSAL IMMUNE RESPONSE, TOLERANCE, AND PROLIFERATION IN MICE COLONIZED BY THE MUCIN-DEGRADER AKKERMANSIA MUCINIPHILA | FRONTIERS IN MICROBIOLOGY | 10.3389/fmicb.2011.00166 | 363 |
| 2011 | THE GENOME OF AKKERMANSIA MUCINIPHILA, A DEDICATED INTESTINAL MUCIN DEGRADER, AND ITS USE IN EXPLORING INTESTINAL METAGENOMES | PLOS ONE | 10.1371/journal.pone.0016876 | 262 |
| 2011 | DO NUTRIENT-GUT-MICROBIOTA INTERACTIONS PLAY A ROLE IN HUMAN OBESITY, INSULIN RESISTANCE AND TYPE 2 DIABETES? | OBESITY REVIEWS | 10.1111/j.1467-789X.2010.00797.x | 211 |
| 2013 | CROSS-TALK BETWEEN AKKERMANSIA MUCINIPHILA AND INTESTINAL EPITHELIUM CONTROLS DIET-INDUCED OBESITY | PROCEEDINGS OF THE NATIONAL ACADEMY OF SCIENCES OF THE UNITED STATES OF AMERICA | 10.1073/pnas.1219451110 | 2827 |
| 2013 | GLYCOBIOME: BACTERIA AND MUCUS AT THE EPITHELIAL INTERFACE | BEST PRACTICE & RESEARCH CLINICAL GASTROENTEROLOGY | 10.1016/j.bpg.2013.03.001 | 150 |
| 2014 | DIFFERENTIAL MODULATION BY AKKERMANSIA MUCINIPHILA AND FAECALIBACTERIUM PRAUSNITZII OF HOST PERIPHERAL LIPID METABOLISM AND HISTONE ACETYLATION IN MOUSE GUT ORGANOIDS | MBIO | 10.1128/mBio.01438-14 | 312 |
| 2015 | AKKERMANSIA MUCINIPHILA ADHERES TO ENTEROCYTES AND STRENGTHENS THE INTEGRITY OF THE EPITHELIAL CELL LAYER | APPLIED AND ENVIRONMENTAL MICROBIOLOGY | 10.1128/AEM.04050-14 | 339 |
| 2015 | AKKERMANSIA MUCINIPHILA AND HELICOBACTER TYPHLONIUS MODULATE INTESTINAL TUMOR DEVELOPMENT IN MICE | CARCINOGENESIS | 10.1093/carcin/bgv120 | 74 |
| 2016 | ADAPTATION OF AKKERMANSIA MUCINIPHILA TO THE OXIC-ANOXIC INTERFACE OF THE MUCUS LAYER | APPLIED AND ENVIRONMENTAL MICROBIOLOGY | 10.1128/AEM.01641-16 | 76 |
| 2016 | CHARACTERIZATION OF OUTER MEMBRANE PROTEOME OF AKKERMANSIA MUCINIPHILA REVEALS SETS OF NOVEL PROTEINS EXPOSED TO THE HUMAN INTESTINE | FRONTIERS IN MICROBIOLOGY | 10.3389/fmicb.2016.01157 | 72 |
| 2016 | AKKERMANSIA GLYCANIPHILA SP NOV., AN ANAEROBIC MUCIN-DEGRADING BACTERIUM ISOLATED FROM RETICULATED PYTHON FAECES | INTERNATIONAL JOURNAL OF SYSTEMATIC AND EVOLUTIONARY MICROBIOLOGY | 10.1099/ijsem.0.001399 | 44 |
| 2016 | INTERACTION OF MOUSE SPLENOCYTES AND MACROPHAGES WITH BACTERIAL STRAINS IN VITRO: THE EFFECT OF AGE IN THE IMMUNE RESPONSE | BENEFICIAL MICROBES | 10.3920/BM2015.0094 | 8 |
| 2017 | A PURIFIED MEMBRANE PROTEIN FROM AKKERMANSIA MUCINIPHILA OR THE PASTEURIZED BACTERIUM IMPROVES METABOLISM IN OBESE AND DIABETIC MICE | NATURE MEDICINE | 10.1038/nm.4236 | 1117 |
| 2017 | PILI-LIKE PROTEINS OF AKKERMANSIA MUCINIPHILA MODULATE HOST IMMUNE RESPONSES AND GUT BARRIER FUNCTION | PLOS ONE | 10.1371/journal.pone.0173004 | 260 |
| 2017 | MICROBIAL METABOLIC NETWORKS AT THE MUCUS LAYER LEAD TO DIET-INDEPENDENT BUTYRATE AND VITAMIN B12 PRODUCTION BY INTESTINAL SYMBIONTS | MBIO | 10.1128/mBio.00770-17 | 207 |
| 2017 | ACTION AND FUNCTION OF AKKERMANSIA MUCINIPHILA IN MICROBIOME ECOLOGY, HEALTH AND DISEASE | BEST PRACTICE & RESEARCH CLINICAL GASTROENTEROLOGY | 10.1016/j.bpg.2017.10.001 | 153 |
| 2017 | GENOME-SCALE MODEL AND OMICS ANALYSIS OF METABOLIC CAPACITIES OF AKKERMANSIA MUCINIPHILA REVEAL A PREFERENTIAL MUCIN-DEGRADING LIFESTYLE | APPLIED AND ENVIRONMENTAL MICROBIOLOGY | 10.1128/AEM.01014-17 | 127 |
| 2017 | MICROBE PROFILE: AKKERMANSIA MUCINIPHILA: A CONSERVED INTESTINAL SYMBIONT THAT ACTS AS THE GATEKEEPER OF OUR MUCOSA | MICROBIOLOGY-SGM | 10.1099/mic.0.000444 | 92 |
| 2017 | IN VITRO COLONISATION OF THE DISTAL COLON BY AKKERMANSIA MUCINIPHILA IS LARGELY MUCIN AND PH DEPENDENT | BENEFICIAL MICROBES | 10.3920/BM2016.0013 | 51 |
| 2017 | ENCAPSULATION OF THE THERAPEUTIC MICROBE AKKERMANSIA MUCINIPHILA IN A DOUBLE EMULSION ENHANCES SURVIVAL IN SIMULATED GASTRIC CONDITIONS | FOOD RESEARCH INTERNATIONAL | 10.1016/j.foodres.2017.09.004 | 44 |
| 2017 | PREPARATION AND PRESERVATION OF VIABLE AKKERMANSIA MUCINIPHILA CELLS FOR THERAPEUTIC INTERVENTIONS | BENEFICIAL MICROBES | 10.3920/BM2016.0096 | 21 |
| 2017 | C4B GENE INFLUENCES INTESTINAL MICROBIOTA THROUGH COMPLEMENT ACTIVATION IN PATIENTS WITH PAEDIATRIC-ONSET INFLAMMATORY BOWEL DISEASE | CLINICAL AND EXPERIMENTAL IMMUNOLOGY | 10.1111/cei.13040 | 17 |
| 2018 | AKKERMANSIA MUCINIPHILA INDUCES GUT MICROBIOTA REMODELLING AND CONTROLS ISLET AUTOIMMUNITY IN NOD MICE | GUT | 10.1136/gutjnl-2017-314508 | 211 |
| 2018 | DECIPHERING THE TROPHIC INTERACTION BETWEEN AKKERMANSIA MUCINIPHILA AND THE BUTYROGENIC GUT COMMENSAL ANAEROSTIPES CACCAE USING A METATRANSCRIPTOMIC APPROACH | ANTONIE VAN LEEUWENHOEK INTERNATIONAL JOURNAL OF GENERAL AND MOLECULAR MICROBIOLOGY | 10.1007/s10482-018-1040-x | 66 |
| 2018 | MODEL-DRIVEN DESIGN OF A MINIMAL MEDIUM FOR AKKERMANSIA MUCINIPHILA CONFIRMS MUCUS ADAPTATION | MICROBIAL BIOTECHNOLOGY | 10.1111/1751-7915.13033 | 29 |
| 2019 | SUPPLEMENTATION WITH AKKERMANSIA MUCINIPHILA IN OVERWEIGHT AND OBESE HUMAN VOLUNTEERS: A PROOF-OF-CONCEPT EXPLORATORY STUDY | NATURE MEDICINE | 10.1038/s41591-019-0495-2 | 977 |
| 2019 | AKKERMANSIA MUCINIPHILA AMELIORATES THE AGE-RELATED DECLINE IN COLONIC MUCUS THICKNESS AND ATTENUATES IMMUNE ACTIVATION IN ACCELERATED AGING ERCC1-/7 MICE | IMMUNITY & AGEING | 10.1186/s12979-019-0145-z | 99 |
| 2019 | INTESTINAL EPITHELIAL N-ACYLPHOSPHATIDYLETHANOLAMINE PHOSPHOLIPASE D LINKS DIETARY FAT TO METABOLIC ADAPTATIONS IN OBESITY AND STEATOSIS | NATURE COMMUNICATIONS | 10.1038/s41467-018-08051-7 | 92 |
| 2020 | PASTEURIZED AKKERMANSIA MUCINIPHILA INCREASES WHOLE-BODY ENERGY EXPENDITURE AND FECAL ENERGY EXCRETION IN DIET-INDUCED OBESE MICE | GUT MICROBES | 10.1080/19490976.2020.1737307 | 104 |
| 2020 | AKKERMANSIA MUCINIPHILA REDUCES PORPHYROMONAS GINGIVALIS-INDUCED INFLAMMATION AND PERIODONTAL BONE DESTRUCTION | JOURNAL OF CLINICAL PERIODONTOLOGY | 10.1111/jcpe.13214 | 63 |
| 2020 | AKKERMANSIA MUCINIPHILA USES HUMAN MILK OLIGOSACCHARIDES TO THRIVE IN THE EARLY LIFE CONDITIONS IN VITRO | SCIENTIFIC REPORTS | 10.1038/s41598-020-71113-8 | 57 |
| 2020 | AKKERMANSIA MUCINIPHILA EXERTS LIPID-LOWERING AND IMMUNOMODULATORY EFFECTS WITHOUT AFFECTING NEOINTIMA FORMATION IN HYPERLIPIDEMIC APOE*3-LEIDEN.CETP MICE | MOLECULAR NUTRITION & FOOD RESEARCH | 10.1002/mnfr.201900732 | 32 |
| 2020 | PASTEURIZED AKKERMANSIA MUCINIPHILA PROTECTS FROM FAT MASS GAIN BUT NOT FROM BONE LOSS | AMERICAN JOURNAL OF PHYSIOLOGY-ENDOCRINOLOGY AND METABOLISM | 10.1152/ajpendo.00425.2019 | 24 |
| 2021 | GENOMIC DIVERSITY AND ECOLOGY OF HUMAN-ASSOCIATED AKKERMANSIA SPECIES IN THE GUT MICROBIOME REVEALED BY EXTENSIVE METAGENOMIC ASSEMBLY | GENOME BIOLOGY | 10.1186/s13059-021-02427-7 | 46 |
| 2021 | BENEFICIAL EFFECTS OF AKKERMANSIA MUCINIPHILA ARE NOT ASSOCIATED WITH MAJOR CHANGES IN THE CIRCULATING ENDOCANNABINOIDOME BUT LINKED TO HIGHER MONO-PALMITOYL-GLYCEROL LEVELS AS NEW PPARΑ AGONISTS | CELLS | 10.3390/cells10010185 | 35 |
| 2021 | SERUM METABOLITE PROFILING YIELDS INSIGHTS INTO HEALTH PROMOTING EFFECT OF A. MUCINIPHILA IN HUMAN VOLUNTEERS WITH A METABOLIC SYNDROME | GUT MICROBES | 10.1080/19490976.2021.1994270 | 18 |
| 2021 | NEXT-GENERATION THERAPEUTIC BACTERIA FOR TREATMENT OF OBESITY, DIABETES, AND OTHER ENDOCRINE DISEASES | BEST PRACTICE & RESEARCH CLINICAL ENDOCRINOLOGY & METABOLISM | 10.1016/j.beem.2021.101504 | 15 |
| 2021 | A CONTINUOUS BATTLE FOR HOST-DERIVED GLYCANS BETWEEN A MUCUS SPECIALIST AND A GLYCAN GENERALIST IN VITRO AND IN VIVO | FRONTIERS IN MICROBIOLOGY | 10.3389/fmicb.2021.632454 | 13 |
| 2021 | GENOMIC CONVERGENCE BETWEEN AKKERMANSIA MUCINIPHILA IN DIFFERENT MAMMALIAN HOSTS | BMC MICROBIOLOGY | 10.1186/s12866-021-02360-6 | 10 |
| 2022 | AKKERMANSIA MUCINIPHILA REDUCES PERITONITIS AND IMPROVES INTESTINAL TISSUE WOUND HEALING AFTER A COLONIC TRANSMURAL DEFECT BY A MYD88-DEPENDENT MECHANISM | CELLS | 10.3390/cells11172666 | 6 |
| 2022 | COMPARATIVE GENOMICS AND PHYSIOLOGY OF AKKERMANSIA MUCINIPHILA ISOLATES FROM HUMAN INTESTINE REVEAL SPECIALIZED MUCOSAL ADAPTATION | MICROORGANISMS | 10.3390/microorganisms10081605 | 4 |
| 2022 | CAMU-CAMU REDUCES OBESITY AND IMPROVES DIABETIC PROFILES OF OBESE AND DIABETIC MICE: A DOSE-RANGING STUDY | METABOLITES | 10.3390/metabo12040301 | 4 |
| 2022 | FOOD REWARD ALTERATIONS DURING OBESITY ARE ASSOCIATED WITH INFLAMMATION IN THE STRIATUM IN MICE: BENEFICIAL EFFECTS OF AKKERMANSIA MUCINIPHILA | CELLS | 10.3390/cells11162534 | 3 |
| 2022 | DIFFERENTIAL EFFECTS OF AKKERMANSIA-ENRICHED FECAL MICROBIOTA TRANSPLANT ON ENERGY BALANCE IN FEMALE MICE ON HIGH-FAT DIET | FRONTIERS IN ENDOCRINOLOGY | 10.3389/fendo.2022.1010806 | 2 |
| 2022 | PEPTIDOGLYCAN FROM AKKERMANSIA MUCINIPHILA MUCT: CHEMICAL STRUCTURE AND IMMUNOSTIMULATORY PROPERTIES OF MUROPEPTIDES | GLYCOBIOLOGY | 10.1093/glycob/cwac027 | 0 |
| 2023 | PASTEURIZED AKKERMANSIA MUCINIPHILA IMPROVES GLUCOSE METABOLISM IS LINKED WITH INCREASED HYPOTHALAMIC NITRIC OXIDE RELEASE | HELIYON | 10.1016/j.heliyon.2023.e18196 | 0 |
